# Supplementary material for: Organization and dynamics of the cortical complexes controlling insulin secretion in β-cells
Source: J Cell Sci. 2022 Feb 3;135(3):jcs259430. doi: 10.1242/jcs.259430 (PMC8918791; doi:10.1242/jcs.259430)
Supplement: Supplementary information [file joces-135-259430-s1.pdf]

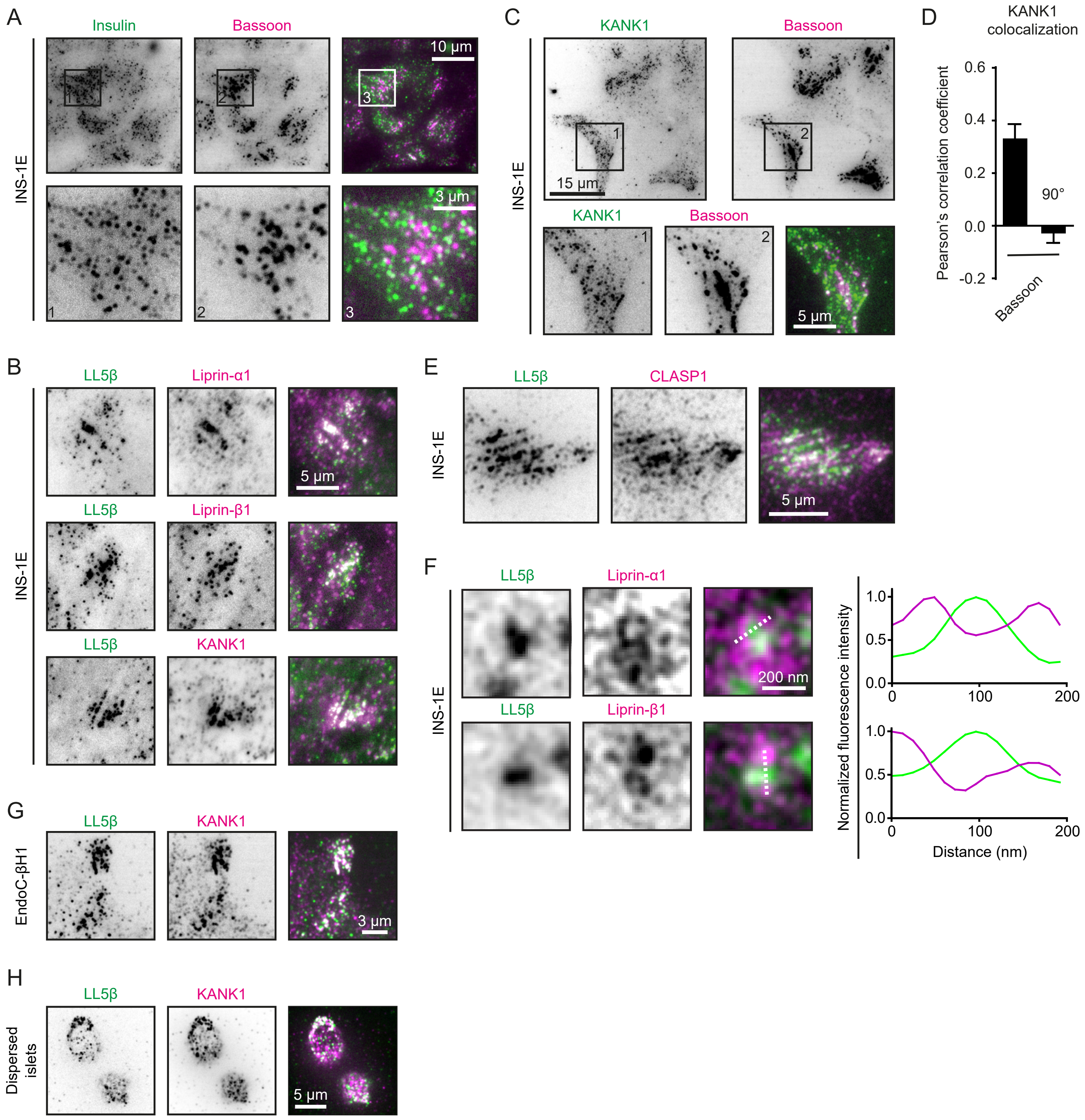

Fig. S1. Organization of the insulin secretion sites in INS-1E cells.
(A) Staining for insulin (green) and Bassoon (magenta) in INS-1E cells imaged with TIRFM. (B) Staining for LL5β (green) and liprin-α1, liprin-β1 and KANK1 (magenta) in INS-1E cells imaged with TIRFM. (C) Staining for KANK1 (green) and Bassoon (magenta) in INS-1E cells imaged with TIRFM. (D) Quantification of colocalization between KANK1 and Bassoon in INS-1E cells. Analysis and display as in Fig. 1B. n=12 ROIs. (E) Staining for LL5β (green) and CLASP1 (magenta) in INS-1E cells imaged with TIRFM. (F) Stimulated Emission Depletion (STED) microscopy images of LL5β (green) and liprin-α1 and liprin-β1 (magenta) in INS-1E cells. Intensity profiles along dotted lines are plotted in graphs. (G) Staining for LL5β (green) and KANK1 (magenta) in EndoC-βH1 cells imaged with TIRFM. (H) Staining for LL5β (green) and KANK1 (magenta) in dispersed human pancreatic islets imaged with TIRFM.

Journal of Cell Science • Supplementary information

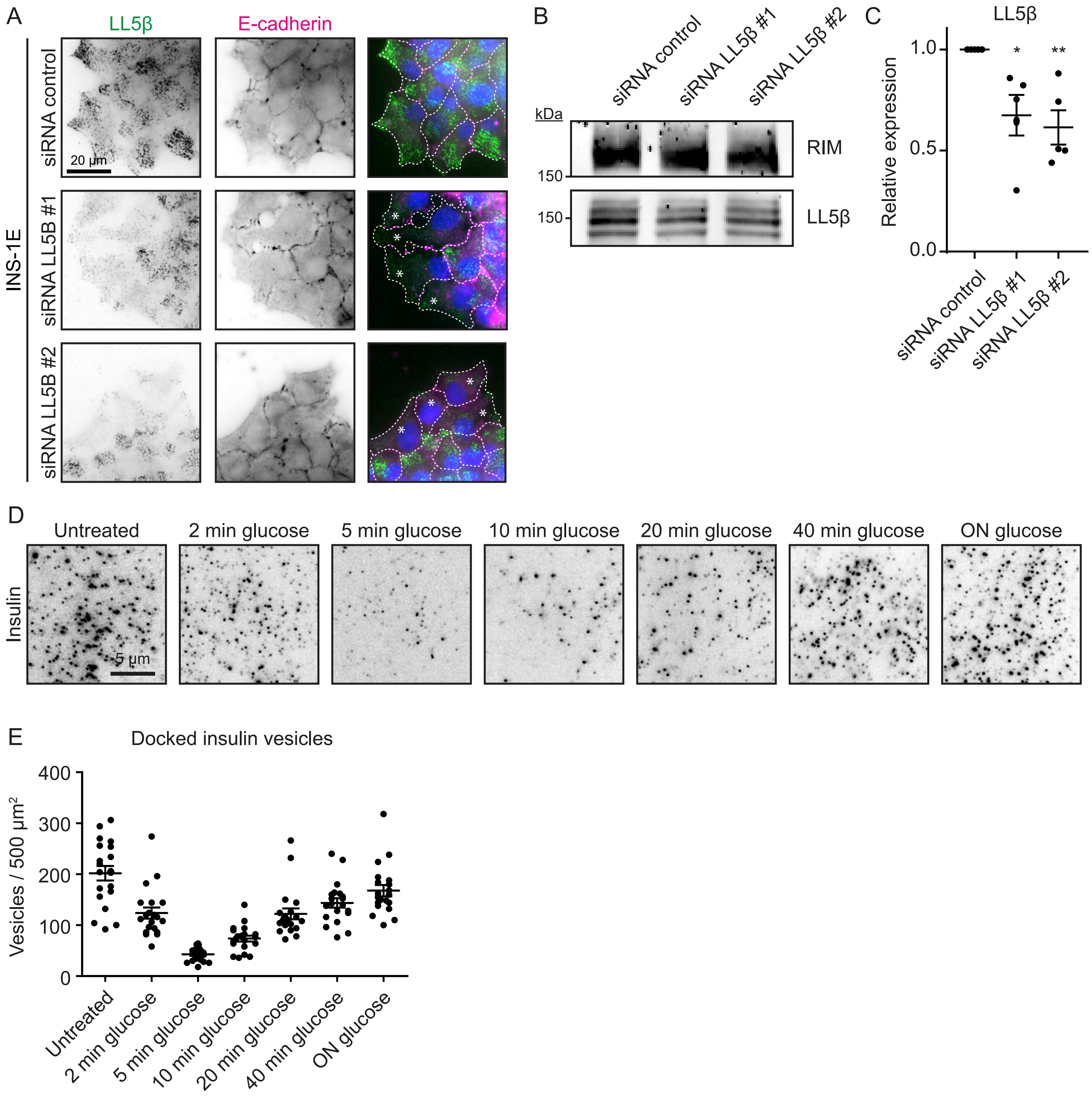

Fig. S2. LL5β knock-down and glucose stimulated insulin secretion in INS-1E cells.
(A) Staining for LL5β (green), E-cadherin (magenta) and DNA (blue) in INS-1E cells transfected with control siRNA or siRNAs against LL5β imaged with widefield microscopy. White asterisks indicate cells with LL5β knock-down. (B) Western blot analysis of RIM and LL5β expression in INS-1E cells treated with siRNAs as indicated. (C) Quantification of LL5β expression based on Western blot analysis as shown in A. *$p<0.1$; **$p<0.01$; one-way ANOVA followed by Dunnett´s post-test. Single data points are plotted. Horizontal line, mean; error bars, S.E.M. For all conditions, n=5 ROIs. (D) Staining for insulin in INS-1E cells starved with 2 mM glucose for 4 hours followed by 25 mM glucose stimulation for indicated times and imaged with TIRFM. (E) Quantification of docked insulin vesicles in INS-1E cells treated and stained as in C. Analysis and display as in Fig. 2G. For all conditions, n=20 ROIs.

Journal of Cell Science • Supplementary information

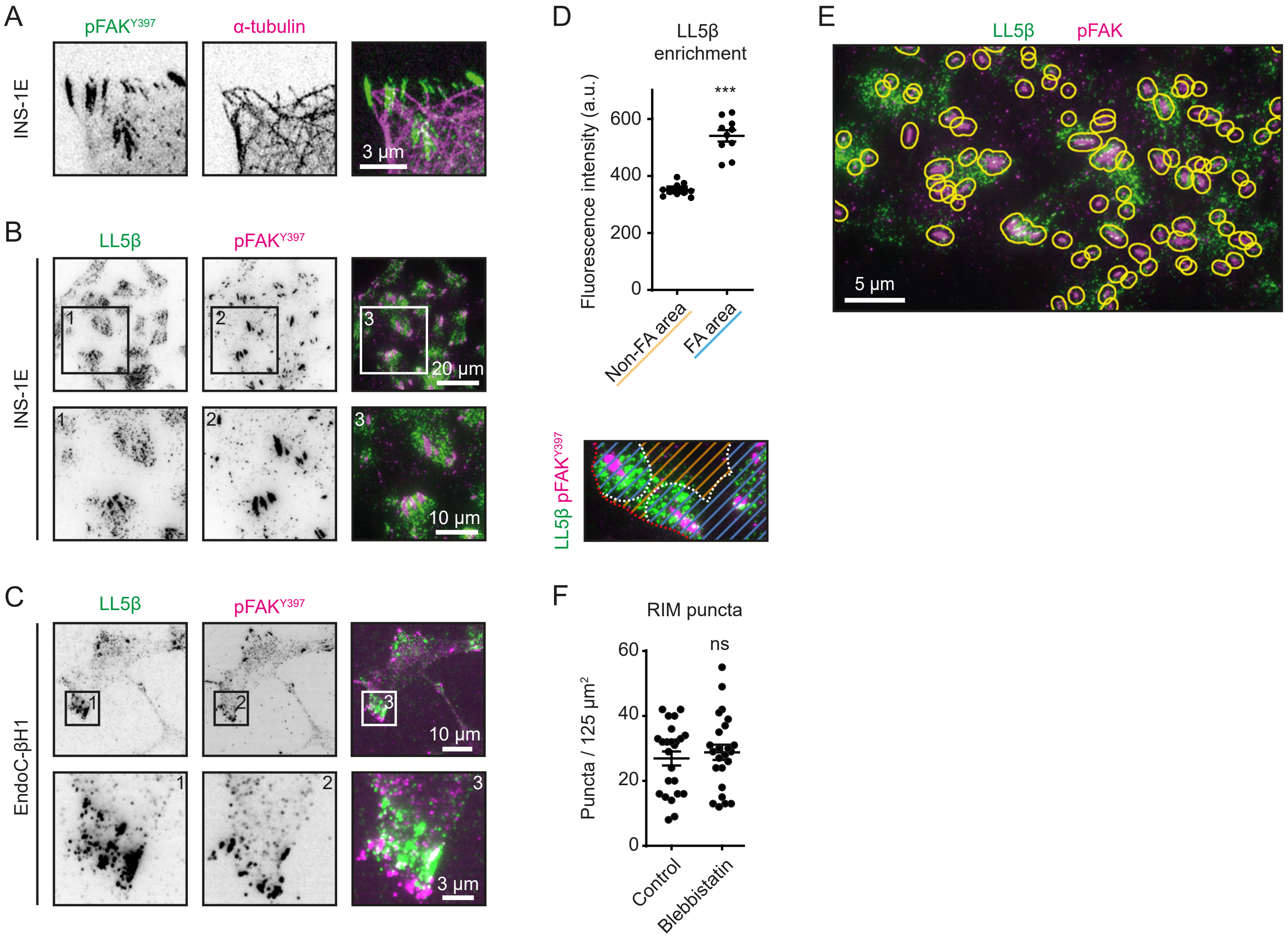

Fig. S3. Analysis of the distribution of cortical secretion complexes.
(A) Stimulated Emission Depletion (STED) microscopy images of phosphorylated FAK ($pFAK^{Y397}$, green) and α-tubulin (magenta) in INS-1E cells. (B) Staining for LL5β (green) and phosphorylated FAK ($pFAK^{Y397}$, magenta) in INS-1E cells imaged with TIRFM. (C) Staining for LL5β (green) and phosphorylated FAK ($pFAK^{Y397}$, magenta) in EndoC-βH1 cells imaged with TIRFM. (D) Quantification of LL5β localization relative to focal adhesions in INS-1E cells. Definition of analyzed cell areas are indicated in scheme. Non-focal adhesion area (orange stripes); focal adhesion area (blue stripes); cell boundary (red dotted line). Single data points are plotted. For both conditions, n=10 focal adhesions; ***$p<0.001$; Mann-Whitney U-test; error bars, S.E.M. (E) Analysis example of LL5β (green) localization relative to focal adhesions (pFAKY397, magenta) in INS-1E cells. Yellow lines indicate areas in which LL5β fluorescence signal was quantified in Fig. 3F. (F). Quantification of the numbers of RIM puncta in INS-1E cells treated and stained as in Fig. 3H. Analysis and display as in Fig. 2C. For both conditions, n=24 ROIs.

Journal of Cell Science • Supplementary information

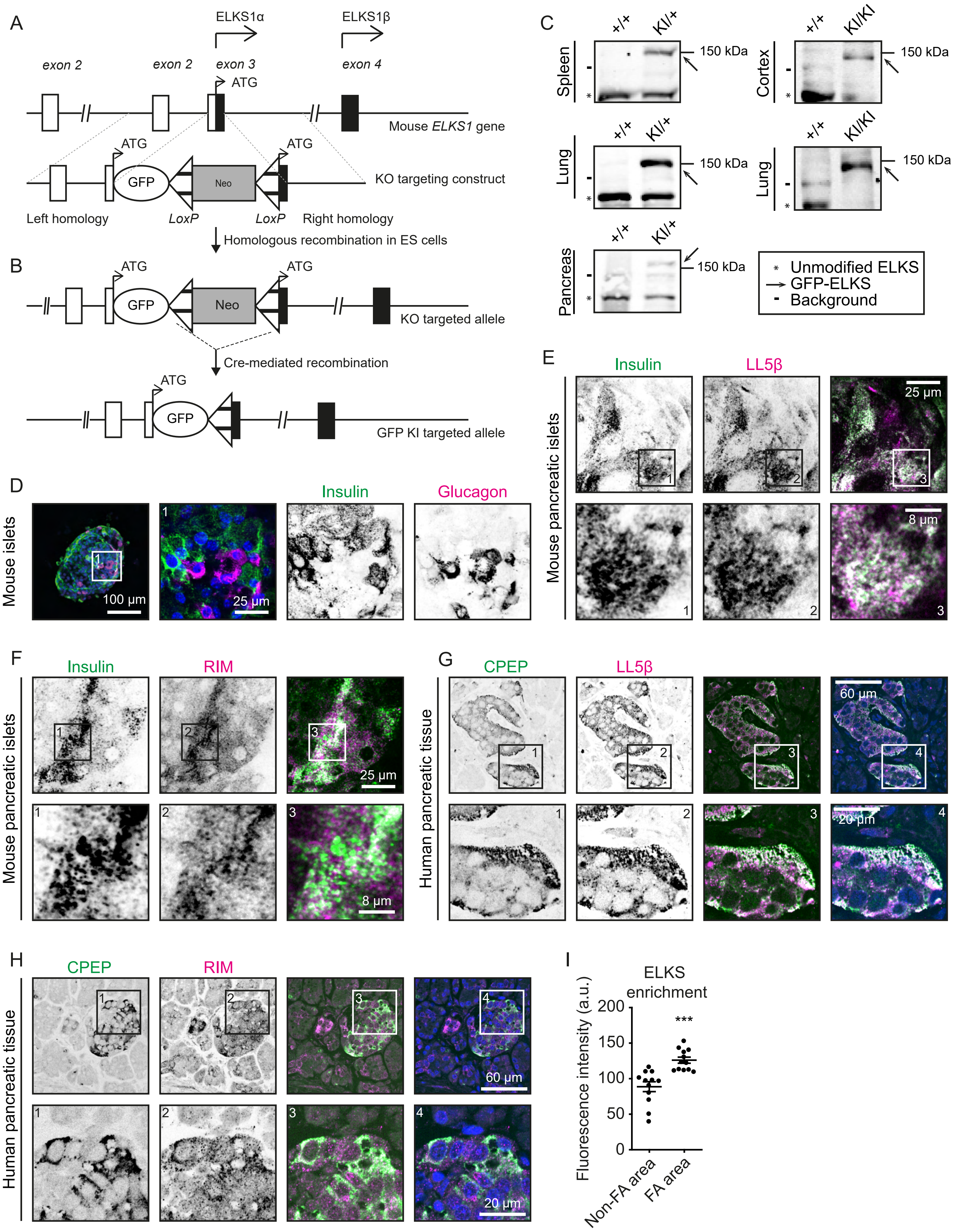

Journal of Cell Science • Supplementary information

Fig. S4. Generation and characterization of GFP-ELKS knock-in mouse line.
(A) Schematic representation of the ELKS knockout (KO) targeting construct. The top line represents the first four exons of ELKS1 gene on mouse chromosome 6. The bottom line represents the ELKS knockout targeting construct containing GFP, the neomycin resistance cassette (NEO) and two LoxP sites (represented by triangles) flanking both sides of NEO. The KO targeting construct has been inserted into exon 3. (B) Schematic representation of the ELKS KO allele and the generation of GFP-ELKS knock-in (KI) targeted allele. The top line shows the ELKS KO targeted allele; after Cre-mediated recombination, the GFP-ELKS KI targeted allele is generated (bottom). (C) Western blot analysis of the indicated mouse tissues with ELKS antibodies. The bands corresponding to unmodified ELKS are indicated by asterisks, GFP-ELKS by arrows, and background bands by lines. +/+, wild type; KI/+, heterozygous GFP-ELKS knock-in; KI/KI homozygous GFP-ELKS knock-in. (D) Staining for insulin (green), glucagon (magenta) and DNA (blue) in a wild type mouse pancreatic islet imaged by confocal microscopy. (E) Staining for insulin (green) and LL5β (magenta) in an adherent region of a mouse pancreatic islet imaged by confocal microscopy. (F) Staining for insulin (green) and RIM (magenta) in an adherent region of a mouse pancreatic islet imaged by confocal microscopy. (G) Staining for C-peptide (CPEP, green) and LL5β (magenta) and DNA (blue) in human pancreatic tissue imaged by confocal microscopy. (H) Staining for C-peptide (CPEP, green) and RIM (magenta) and DNA (blue) in human pancreatic tissue imaged by confocal microscopy. (I) Quantification of ELKS localization relative to focal adhesions in INS-1E cells. Analysis and display as in Fig. S3D. For all conditions, n=12 focal adhesions.

Journal of Cell Science • Supplementary information

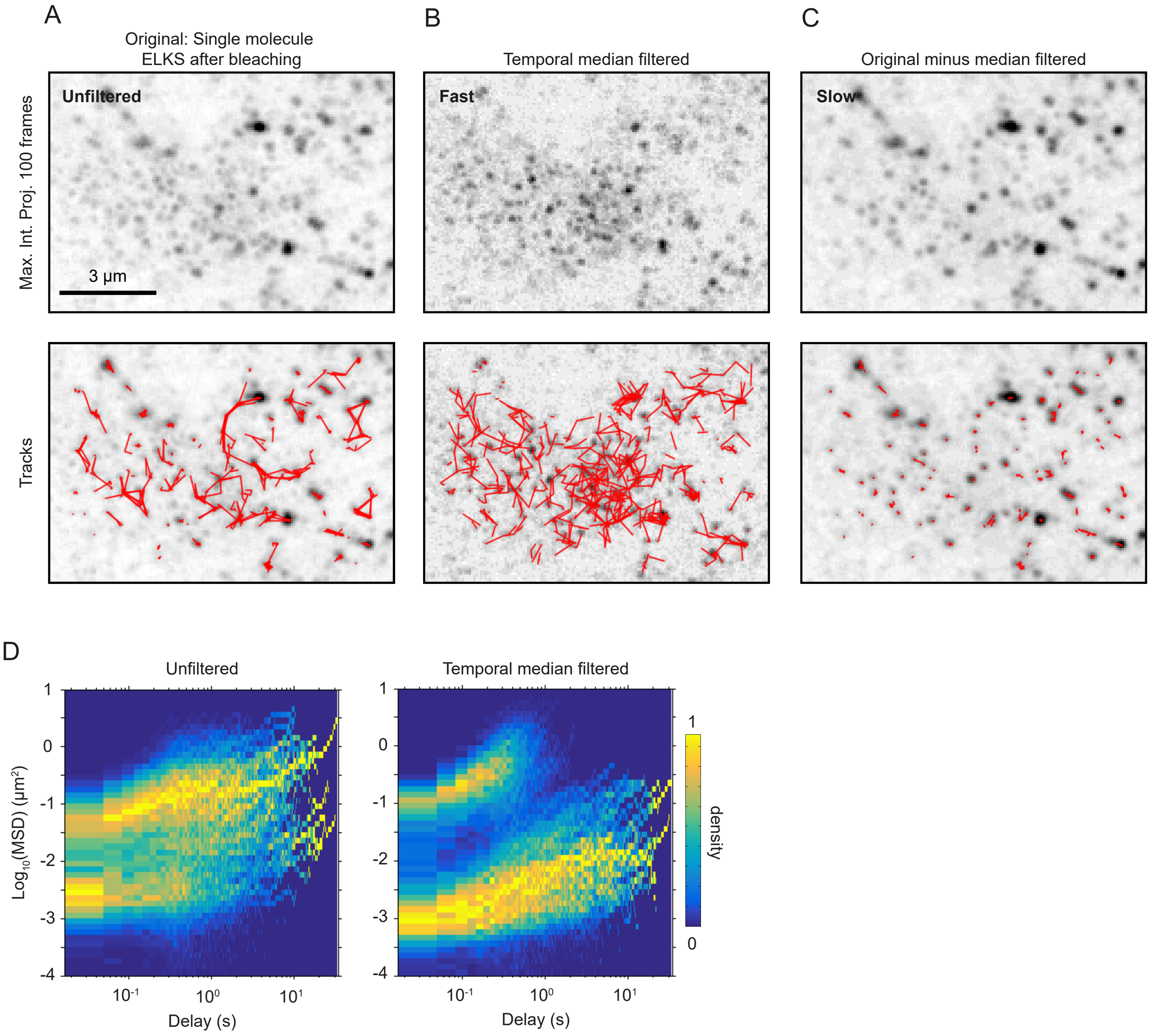

Fig. S5. Single molecule analysis of GFP-ELKS in mouse pancreatic islets.
(A) Representative maximum intensity projection of single GFP-ELKS molecules dynamics (100 frames, 33 ms per frame, top) and the corresponding trajectories (bottom). (B) Maximum intensity projection of the movie shown in (A) after application of temporal median filtering with the window size of 15 frames (top) and the corresponding trajectories (bottom). Fast-moving fraction of single molecules is highlighted as a result. (C) Maximum intensity projection of the result of frame-by-frame and per pixel subtraction of movie shown in B from the movie shown in A (top) and corresponding trajectories (bottom). Slow-moving fraction of single molecules is highlighted as a result. (D) Heatmap (3D histogram) of MSD values for the trajectories of single GFP-ELKS molecules, tracked with and without temporal median filtering. Histogram values are normalized by the maximum value of each column, corresponding to each time delay bin.

Journal of Cell Science • Supplementary information

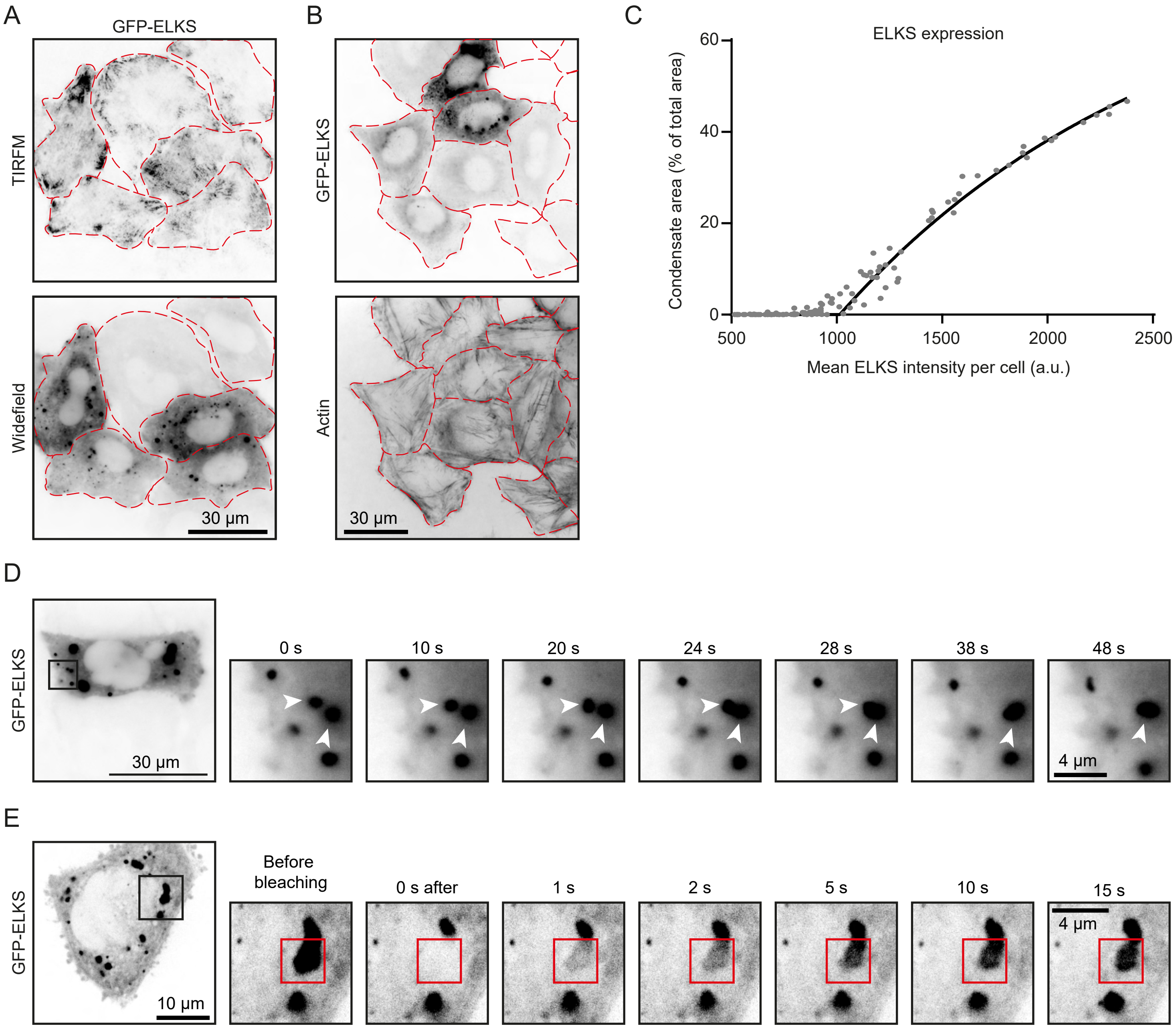

Fig. S6. Analysis of condensates in HeLa cells overexpressing GFP-ELKS.
(A) Live HeLa cells with transient overexpression of GFP-ELKS imaged by TIRFM (top) and widefield microscopy (bottom). Red dotted lines indicate cell borders. (B) Staining for actin (bottom) in HeLa cells transiently overexpressing GFP-ELKS imaged by widefield microscopy. Red dotted lines indicate cell borders. (C) Quantification of GFP-ELKS expression in HeLa cells treated and stained as in (B). Data are plotted as percentage of cell area occupied by condensates against the mean GFP-ELKS intensity per cell. Dots represent single data points; line shows non-linear fit; n=115 cells. (D) Live HeLa cells with transient overexpression of GFP-ELKS imaged by widefield microscopy. White arrowheads indicate fusion of condensates. (E) FRAP in HeLa cells transiently overexpressing GFP-ELKS imaged by confocal microscopy. Red squares indicate the photobleached region.

Journal of Cell Science • Supplementary information

Table S1. Average FRAP curves fitting parameters

Fitted value ± error of fit / Condition	Plateau (exchangeable fraction)	Fast halftime (min)	Slow halftime (min)	Percent fast
Low glucose	0.42 ± 0.02	0.93 ± 0.37	4.8 ± 1.9	40 ± 16
High glucose	0.65 ± 0.03	0.67 ± 0.26	5.5 ± 1.1	24 ± 6

Table S2. Key Resources Table

REAGENT or RESOURCE	SOURCE	IDENTIFIER	ANTIBODY DILUTION IF
Antibodies			
Mouse anti-LL5β	Dr. J. Sanes ; (Kishi et al., 2005)	N/A	1:200
Mouse anti-Bassoon (SAP7F407)	Enzo Life Sciences	Cat#ADI-VAMPS003; RRID: AB_10618753	1:200
Mouse anti-paxillin (Clone 165)	BD Biosciences	Cat# 610619; RRID: AB_397951	1:100
Mouse anti-glucagon (Clone K79bB10)	Abcam	Cat# ab10988; RRID:AB_297642	1:100
Rabbit anti-LL5β	(Lansbergen et al., 2006)	N/A	1:200
Rabbit anti-ERC1	Proteintech Group	Cat# 22211-1-AP; RRID:AB_11232409	1:200
Rabbit anti-ERC1 c-terminus	Dr. F. Melchior; (Grigoriev et al., 2011)	N/A	1:200
Rabbit anti-liprin-α1	(Spangler et al., 2011)	N/A	1:100
Rabbit anti-liprin-β1	(van der Vaart et al., 2013)	N/A	1:50
Rabbit anti-KANK1	Sigma-Aldrich	Cat# HPA005539; RRID:AB_1078164	1:400
Rabbit anti-RIM1/2	Synaptic Systems	Cat# 140 203; RRID:AB_887775	1:500
Rabbit anti-CLASP1	(Mimori-Kiyosue et al., 2005)	N/A	1:400
Rabbit anti-PhosphoFAK (Tyr397) (pFAK) (31H5L17)	Thermo Fisher Scientific	Cat# 700255; RRID:AB_2532307	1:300
Rabbit anti-E-cadherin	Gift from A. Yap	N/A	1:1000
Rat Anti-Mouse CD144 (VE-cadherin)	BD Biosciences	Cat# 555289; RRID:AB_395707	1:100
Rat anti-tyrosinated α-tubulin (Clone YL1/2)	Abcam	Cat# ab6160; RRID:AB_305328	1:300
Guinea pig anti-insulin	Dako	Cat# A0564; RRID:AB_10013624	1:500
Guinea pig anti C-peptide	Abcam	Cat# ab30477, RRID:AB_726924	1:100
Alexa Fluor 488 Phalloidin	Thermo Fisher Scientific	Cat# A12379; RRID:AB_2315147	1:200
Alexa Fluor 594 Phalloidin	Thermo Fisher Scientific	Cat# A12381; RRID:AB_2315633	1:200
Alexa Fluor 647 Phalloidin	Thermo Fisher Scientific	Cat# A22287; RRID:AB_2620155	1:100
Alexa488 Goat anti-Mouse IgG, highly cross-adsorbed	Thermo Fisher Scientific	Cat# A-11001; RRID:AB_2534069	1:300
Alexa594 Goat anti-Mouse IgG, highly cross-adsorbed	Thermo Fisher Scientific	Cat# R37121; RRID:AB_2556549	1:300
Alexa488 Goat anti-rabbit IgG, highly cross-adsorbed	Thermo Fisher Scientific	Cat# A-11034; RRID:AB_2576217	1:300
Alexa594 Goat anti-rabbit IgG, highly cross-adsorbed	Thermo Fisher Scientific	Cat# R37117; RRID:AB_2556545	1:300
Alexa488 Goat anti-rat IgG, highly cross-adsorbed	Thermo Fisher Scientific	Cat# A-11006; RRID:AB_2534074	1:300

Journal of Cell Science • Supplementary information

Alexa594 Goat anti-rat IgG, highly cross-adsorbed	Thermo Fisher Scientific	Cat# A-11007; RRID:AB_10561522	1:300
Alexa488 Goat anti-guinea pig IgG, highly cross-adsorbed	Thermo Fisher Scientific	Cat# A-11073; RRID:AB_2534117	1:300
Alexa594 Goat anti-guinea pig IgG, highly cross-adsorbed	Thermo Fisher Scientific	Cat# A-11076; RRID:AB_2534120	1:300
IRDye 800CW Goat anti-Mouse IgG	Li-cor Biosciences	Cat# 925-32210; RRID:AB_2687825	1:15000
IRDye 800CW Goat anti-Rabbit IgG	Li-cor Biosciences	Cat# 925-32211; RRID:AB_2651127	1:15000
IRDye 680LT Goat anti-Mouse IgG	Li-cor Biosciences	Cat# 925-68020; RRID:AB_2687826	1:15000
IRDye 680LT Goat anti-Rabbit IgG	Li-cor Biosciences	Cat# 925-68021; RRID:AB_2713919	1:15000
Anti-mouse-D1	Ultivue	N/A	1:100
Anti-rabbit-D2	Ultivue	N/A	1:100
Chemicals, Peptides, and Recombinant Proteins			
Blebbistatin	Enzo Life Sciences	Cat# BML-EI315-0025	
LifeAct-mNeonGreen	(Tas et al., 2018)	N/A	
Experimental Models: Cell Lines			
Rat: INS-1E line	(Asfari et al., 1992)	RRID:CVCL_0351	
Human: EndoC-βH1 line	(Ravassard et al., 2011)	RRID:CVCL_L909	
Human: HeLa cell line	JCRB9004	RRID:CVCL_0030	
Experimental Models: Organisms/Strains			
Mouse: C57BL/6	Charles River	C57Bl6/NCrl	
Mouse: GFP-ELKS KI/KI	This paper	NCBI Gene: 111173	
Oligonucleotides			
siRNA targeting sequence LL5β #1: GGAGATTCTAGATCATCTA	(Lansbergen et al., 2006)	N/A	
siRNA targeting sequence LL5β #2: GGATCTACCTCACAGCCTA	This paper	N/A	
siRNA control targeting luciferase: CGTACGCGGAATACTTCGA	(Bouchet et al., 2016b)	N/A	
Imager strand I2-560	Ultivue	N/A	
Imager strand I1-650	Ultivue	N/A	
Software and Algorithms			
GraphPad Prism 9	GraphPad Software		
Metamorph Version 7.8	Molecular Devices		
ImageJ 1.50b			
MATLAB R2011b	MathWorks		

Journal of Cell Science • Supplementary information

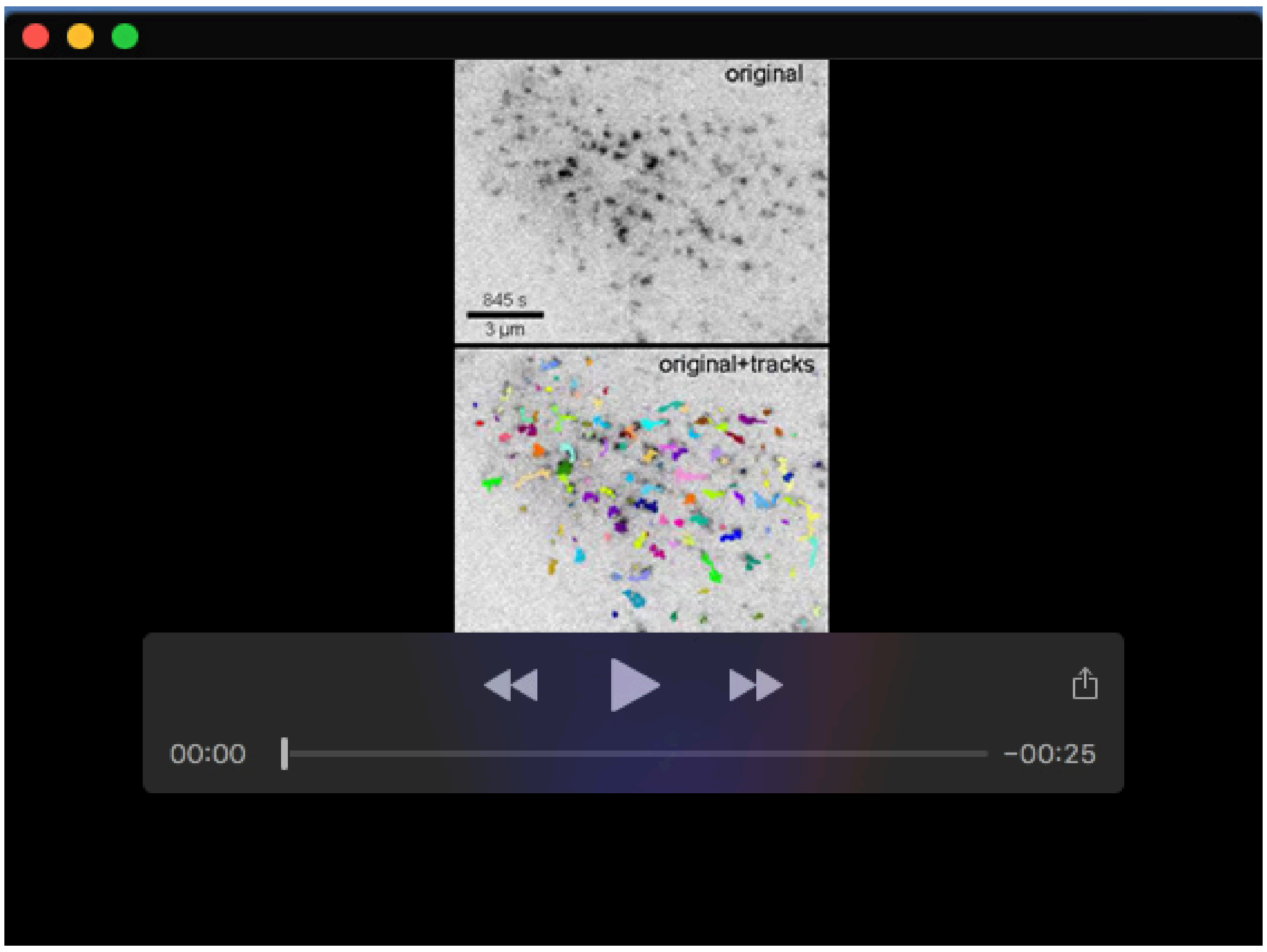

Movie 1. Dynamics GFP-ELKS clusters in an isolated mouse pancreatic islet. An example TIRFM acquisition of GFP-ELKS clusters (top) and the corresponding overlay of trajectories (bottom).

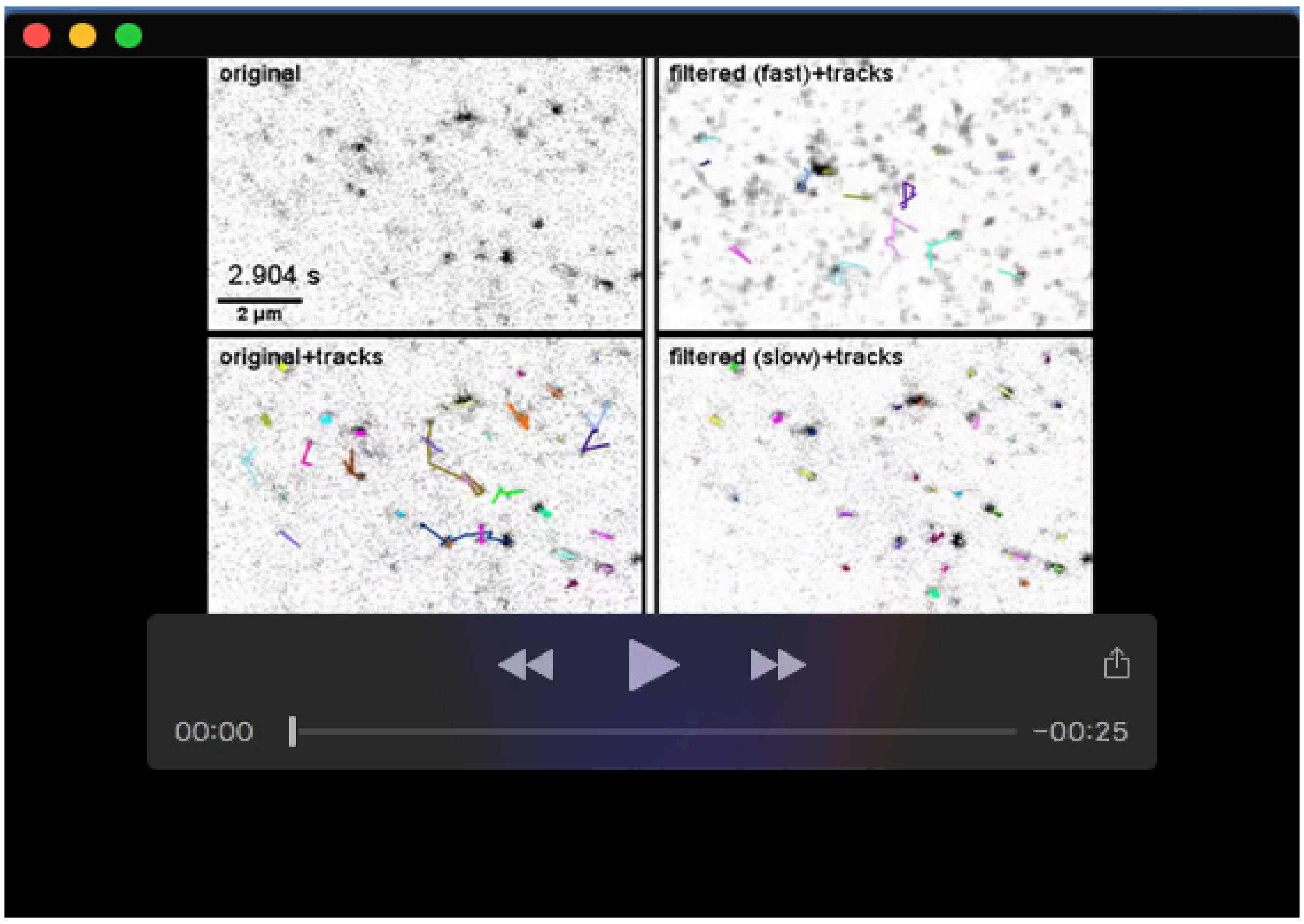

Movie 2. Dynamics of single GFP-ELKS molecules in an isolated mouse pancreatic islet. An example TIRFM acquisition of single GFP-ELKS molecules (top left) and the corresponding overlay of trajectories (bottom left). Right panels illustrate the temporal median filtering method, the splitting of the acquisition into fast (top) and slow (bottom) components.

Journal of Cell Science • Supplementary information

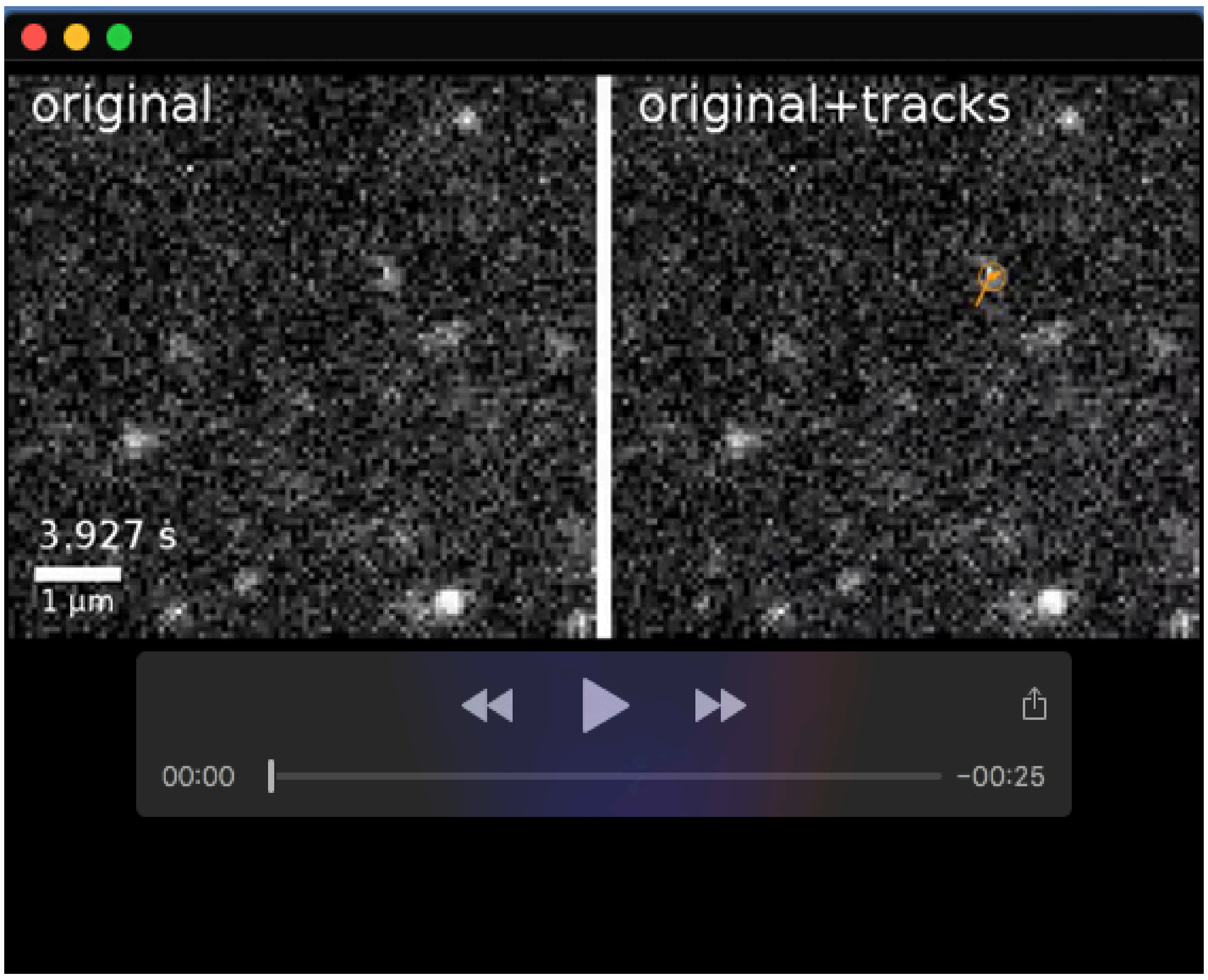

Movie 3. Transitions of single GFP-ELKS molecules between diffusive and tethered states. An example TIRFM acquisition of single GFP-ELKS molecules (left) and the corresponding overlay of trajectories (right).

Journal of Cell Science • Supplementary information
