# Supplementary material for: Organization and dynamics of the cortical complexes controlling insulin secretion in β-cells
Source: J Cell Sci. 2022 Feb 3;135(3):jcs259430. doi: 10.1242/jcs.259430 (PMC8918791; doi:10.1242/jcs.259430)
Supplement: Reviewer comments [file joces-135-259430_review_history.pdf]

Organization and dynamics of the cortical complexes controlling insulin secretion in ß-cells

Ivar Noordstra, Cyntha M. van den Berg, Fransje W.J. Boot, Eugene A. Katrukha, Ka Lou Yu, Roderick P. Tas, Sybren Portegies, Bastiaan J. Viergever, Esther de Graaff, Casper C. Hoogenraad, Eelco J.P. Koning, Françoise Carlotti, Lukas C. Kapitein and Anna Akhmanova
DOI: 10.1242/jcs.259430

Editor: Jennifer Lippincott-Schwartz

Review timeline

Original submission:	1 October 2021
Editorial decision:	18 November 2021
First revision received:	20 December 2021
Accepted:	21 December 2021

Original submission

First decision letter

MS ID#: JOCES/2021/259430

MS TITLE: Organization and dynamics of the cortical complexes controlling insulin secretion in Î²-cells

AUTHORS: Ivar Noordstra, Cyntha M. van den Berg, Fransje W.J. Boot, Eugene A. Katrukha, Ka Lou Yu, Roderick P. Tas, Sybren Portegies, Bastiaan J. Viergever, Esther de Graaff, Casper C. Hoogenraad, Eelco J.P. Koning, Françoise Carlotti, Lukas C. Kapitein, and Anna Akhmanova
ARTICLE TYPE: Research Article

We have now reached a decision on the above manuscript.

To see the reviewers' reports and a copy of this decision letter, please go to: https://submit-jcs.biologists.org and click on the 'Manuscripts with Decisions' queue in the Author Area. (Corresponding author only has access to reviews.)

As you will see, the reviewers raise a number of criticisms that prevent me from accepting the paper at this stage. They suggest, however, that a revised version might prove acceptable, if you can address their concerns. If you think that you can deal satisfactorily with the criticisms on revision, I would be pleased to see a revised manuscript. We would then return it to the reviewers.

We are aware that you may be experiencing disruption to the normal running of your lab that makes experimental revisions challenging. If it would be helpful, we encourage you to contact us to discuss your revision in greater detail. Please send us a point-by-point response indicating where you are able to address concerns raised (either experimentally or by changes to the text) and where you will not be able to do so within the normal timeframe of a revision. We will then provide further guidance. Please also note that we are happy to extend revision timeframes as necessary.

Please ensure that you clearly highlight all changes made in the revised manuscript. Please avoid using 'Tracked changes' in Word files as these are lost in PDF conversion.

© 2022. Published by The Company of Biologists under the terms of the Creative Commons Attribution License (https://creativecommons.org/licenses/by/4.0/).

I should be grateful if you would also provide a point-by-point response detailing how you have dealt with the points raised by the reviewers in the 'Response to Reviewers' box. Please attend to all of the reviewers' comments. If you do not agree with any of their criticisms or suggestions please explain clearly why this is so.

Reviewer 1

Advance summary and potential significance to field

This paper presents some new and interesting findings regarding the role that integrin-based adhesions play in controlling the spatial organization of glucose-stimulated insulin release. It also addresses the nature of ELKS puncta using photobleaching and single molecule imaging. That data argues that ELKS turnover is driven by its binding and unbinding to low-mobility scaffolds.

Comments for the author

Overall I liked this study. I think the topic is quite interesting, and the paper presents some new and interesting findings regarding the role that integrin-based adhesions play in controlling the spatial organization of glucose-stimulated insulin release. I found most of the data convincing. That said, I must confess that while I followed the arguments based on photobleaching and single-molecule imaging that ELKS localization is driven by binding and unbinding to low-mobility scaffolds rather than by liquid-liquid phase separation, I might not be the best person to vet those results!

One significant experimental concern relates to the LL5B knockdown data.
Regarding this, the text reads “we depleted LL5ß in INS-1E cells using two different siRNAs and observed a 30-40% reduction in the LL5ß signal on Western blots (Fig. S2A,B). Immunofluorescence cell staining showed that LL5ß-positive puncta were almost completely lost in ~30-40% of the cells (Fig. 2A,B).” To me these results raise a couple of questions and issues. If all the cells had on average ~30-40% less LL5B, they why did only ~30-40% of cells exhibit a near complete loss of LL5B puncta? Or is it that ~30-40% of the cells had near complete knockdown of LL5B, and it was these cells that exhibited a near complete loss of LL5B? If it’s the latter, the authors should show on an individual cell basis that the loss of puncta correlates with the loss of LL5B protein (by IF). My uncertainty about all this made it hard to be sure about the subsequent data on insulin secretion using the knockdown cells. Also, given the modest degree of knockdown, it seems a rescue experiment would be needed to be sure the effects seen are really due to LL5B knockdown.

Minor concerns:
On page 7, the text reads “We therefore co-stained INS-1E cells for insulin and RIM, and analyzed the cells with dispersed RIM puncta, as we observed that such cells were strongly depleted of LL5ß (Fig. 2A).” Isn’t the data on dispersal of RIM puncta shown in Figure 2D?
On page 10, the authors state “These small clusters were distributed non-homogeneously, often showing local enrichment areas. Such areas often localized around focal adhesions at the base of stress fibers (Fig. 4D), supporting the findings described above.” Can they provide some quantification of this data like they did for other, similar experiments?
On page 13, the authors say in the first sentence in the Discussion “controlling the very rapid Ca2+-regulated neurotransmitter secretion”. I think they might need a reference here since they did not study this in their paper.
Can the authors add statistical analysis for Figure 2D?
In Figure 3A and elsewhere (text, figure legends), please specify the phosphorylation of FAK.
It is pretty hard to see any differences in Figure 3F. Perhaps a graph like in Figure S3D would be more helpful.

© 2022. Published by The Company of Biologists under the terms of the Creative Commons Attribution License (https://creativecommons.org/licenses/by/4.0/).

Reviewer 2

Advance summary and potential significance to field

Noordstra and van den Berge et al. present a thorough data sets to support the conclusions that have made in this manuscript. The experiments are presented with the proper controls. I particularly found the evidence that LLPS is not a major mechanism convincing.

Comments for the author

The only suggestion that I have at this time that will make this manuscript harder is for the authors to repeat the original over-expression experiments to test if ELKS forms liquid-like droplets. If it is from over expression, the authors should be able to separate exogenously expressing beta cells into low and high expressing groups to show if more ELKS makes liquid droplet-like structures appear. If this is the case, then much of the experiments in the LLPS may need to be re-examined.

First revision

Author response to reviewers' comments

We thank the reviewers for their supportive feedback. We have revised the manuscript in light of their comments, and textual changes are indicated in blue in the Revised manuscript.

Reviewer 1 Advance Summary and Potential Significance to Field:

This paper presents some new and interesting findings regarding the role that integrin-based adhesions play in controlling the spatial organization of glucose-stimulated insulin release. It also addresses the nature of ELKS puncta using photobleaching and single molecule imaging. That data argues that ELKS turnover is driven by its binding and unbinding to low-mobility scaffolds.

Reviewer 1 Comments for the Author:

Overall, I liked this study. I think the topic is quite interesting, and the paper presents some new and interesting findings regarding the role that integrin-based adhesions play in controlling the spatial organization of glucose-stimulated insulin release. I found most of the data convincing. That said, I must confess that while I followed the arguments based on photobleaching and single-molecule imaging that ELKS localization is driven by binding and unbinding to low-mobility scaffolds rather than by liquid-liquid phase separation, I might not be the best person to vet those results!

One significant experimental concern relates to the LL5B knockdown data. Regarding this, the text reads "we depleted LL5β in INS-1E cells using two different siRNAs and observed a 30-40% reduction in the LL5β signal on Western blots (Fig. S2A,B). Immunofluorescence cell staining showed that LL5β-positive puncta were almost completely lost in ~30-40% of the cells (Fig. 2A,B)." To me, these results raise a couple of questions and issues. If all the cells had on average ~30-40% less LL5B, they why did only ~30-40% of cells exhibit a near complete loss of LL5B puncta? Or is it that ~30-40% of the cells had near complete knockdown of LL5B, and it was these cells that exhibited a near complete loss of LL5B? If it's the latter, the authors should show on an individual cell basis that the loss of puncta correlates with the loss of LL5B protein (by IF). My uncertainty about all this made it hard to be sure about the subsequent data on insulin secretion using the knockdown cells. Also, given the modest degree of knockdown, it seems a rescue experiment would be needed to be sure the effects seen are really due to LL5B knockdown.

Reply: We confirm that the latter statement of the reviewer is correct. ~30-40% of the cells had a near complete knockdown of LL5β. As a result, these cells exhibit a near complete loss of LL5β puncta. To clarify this point, and as the reviewer suggests, we included an immunofluorescence staining of LL5β and E-cadherin in LL5β knockdown cells (Fig S2A). As E- cadherin has been shown

© 2022. Published by The Company of Biologists under the terms of the Creative Commons Attribution License (https://creativecommons.org/licenses/by/4.0/).

to mediate homophilic cell adhesion between B-cells (Carvell et al., Cell Physiol Biochem. 2007;20(5):617-26), it can be used to define cell borders, which allows for analysis on an individual cell basis. This experiment clearly shows that some cells exhibit a near complete loss of LL5ß puncta (highlighted by *), whereas others still express LL5ß. In contrast, all cells express LL5ß when transfected with control siRNA's.

We agree with the reviewer that rescue experiments would strengthen our observations. Unfortunately, after many attempts with different transfection protocols, we were not able to transfect INS-1E cells with DNA. We believe, however, that the strong correlation between phenotypes and knockdown with 2 independent siRNA's provides a solid and reproducible base for our findings.

Minor concerns:

On page 7, the text reads "We therefore co-stained INS-1E cells for insulin and RIM, and analyzed the cells with dispersed RIM puncta, as we observed that such cells were strongly depleted of LL5β (Fig. 2A)." Isn't the data on dispersal of RIM puncta shown in Figure 2D?

Reply: We thank the reviewer for the correction. The dispersed RIM puncta, shown in figure 2A, is indeed quantified in figure 2D. We now refer to both figure 2A and 2D.

On page 10, the authors state "These small clusters were distributed non-homogeneously, often showing local enrichment areas. Such areas often localized around focal adhesions at the base of stress fibers (Fig. 4D), supporting the findings described above." Can they provide some quantification of this data like they did for other, similar experiments?

Reply: We thank the reviewer for the excellent suggestion and quantified GFP-ELKS distribution relative to focal adhesions as we have done in figure (S3D). The new data is shown in Figure S4I.

On page 13, the authors say in the first sentence in the Discussion "controlling the very rapid Ca2+-regulated neurotransmitter secretion". I think they might need a reference here since they did not study this in their paper.

Reply: We now added the correct references to the discussion.

Can the authors add statistical analysis for Figure 2D?

Reply: We added error bars representing SEMs to figure 2D (and 3I, as this is a similar quantification). To directly compare the groups, we did statistical analysis on weighted averages of the distance between nearest puncta and added additional bar graphs (Fig 2E, 3J) correlated to the distribution graphs.

In Figure 3A and elsewhere (text, figure legends), please specify the phosphorylation of FAK.

Reply: In the methods we mentioned that the antibody recognizes FAK phosphorylation on Tyr397. We now also added this to the text and figure legends.

It is pretty hard to see any differences in Figure 3F. Perhaps a graph like in Figure S3D would be more helpful.

Reply: To improve readability of the graph, and as suggested by the reviewer, we changed Fig 3F to a bar graph.

Reviewer 2 Advance Summary and Potential Significance to Field:

Noordstra and van den Berg et al. present a thorough data sets to support the conclusions that have made in this manuscript. The experiments are presented with the proper controls. I particularly found the evidence that LLPS is not a major mechanism convincing.

© 2022. Published by The Company of Biologists under the terms of the Creative Commons Attribution License (https://creativecommons.org/licenses/by/4.0/).

Reviewer 2 Comments for the Author:

The only suggestion that I have at this time that will make this manuscript harder is for the authors to repeat the original over-expression experiments to test if ELKS forms liquid-like droplets. If it is from over expression, the authors should be able to separate exogenously expressing beta cells into low and high expressing groups to show if more ELKS makes liquid droplet-like structures appear. If this is the case, then much of the experiments in the LLPS may need to be re-examined.

Reply: We thank the reviewer for the positive comments and this very insightful comment. Unfortunately, INS1E cells are very difficult to transfect with plasmids, and therefore, we could not overexpress proteins in this system. Instead, we performed a whole new set of experiments in HeLa cells to address the reviewer's points. We found that at low expression levels, GFP-ELKS is present in peripheral cortical clusters (as we described previously by Lansbergen et al., Dev Cell 2006,11, 21-32; Grigoriev et al., Dev Cell 2007, 13, 305-14, van der Vaart et al., Dev Cell 2013, 27, 145-60), very similar to what we see in ß-cells. In contrast, overexpression of GFP-ELKS resulted in the formation of protein condensates. Importantly, these condensates were located in the cytoplasm and not at the cell cortex, where the physiologically relevant ELKS-containing complexes reside. The number and size of condensates directly correlated with the expression level of GFP-ELKS, indicating that indeed "more ELKS makes liquid droplet-like structures appear", like the reviewer suggests. In addition, we observed typical LLPS condensate-like behaviour like droplet fusion and very rapid recovery after photobleaching after overexpression of GFP-ELKS. Taken together, we show that previously described data on condensate formation by ELKS can be easily reproduced but represent a result of protein overexpression. All these new data are presented in the new Figure S6 and are described in the Results.

<u>Second decision letter</u>

MS ID#: JOCES/2021/259430

MS TITLE: Organization and dynamics of the cortical complexes controlling insulin secretion in Î²-cells

AUTHORS: Ivar Noordstra, Cyntha M. van den Berg, Fransje W.J. Boot, Eugene A. Katrukha, Ka Lou Yu, Roderick P. Tas, Sybren Portegies, Bastiaan J. Viergever, Esther de Graaff, Casper C. Hoogenraad, Eelco J.P. Koning, Françoise Carlotti, Lukas C. Kapitein, and Anna Akhmanova
ARTICLE TYPE: Research Article

I am happy to tell you that your manuscript has been accepted for publication in Journal of Cell Science, pending standard ethics checks.

© 2022. Published by The Company of Biologists under the terms of the Creative Commons Attribution License (https://creativecommons.org/licenses/by/4.0/).
